# Prebending of osteosynthesis plate using 3D printed models to treat symptomatic os acromiale and acromial fracture

**DOI:** 10.1186/s40634-017-0111-7

**Published:** 2017-10-24

**Authors:** Hanne Beliën, Hanne Biesmans, Anny Steenwerckx, Eric Bijnens, Carl Dierickx

**Affiliations:** 1 Bachelor of science in Biomedical Sciences, University Hasselt, Hasselt, Belgium; 2 Orthopedic surgeon Jessa Hospital, Hasselt, Belgium; 3 Radiology department Jessa Hospital, Hasselt, Belgium; 4 Orthopedics, University Hasselt, Hasselt, Belgium

**Keywords:** Os acromiale, Acromial fracture, 3D printing, Osteosynthesis plate, Case report

## Abstract

**Background:**

A symptomatic os acromiale can lead to impingement syndrome and rotator cuff tendinopathy. An acromion fracture is often part of a more complex scapular trauma that needs stabilisation.

**Methods:**

We developed a new technique using a three-dimensional (3D) model and a distal clavicle reconstruction plate to treat os acromiale and acromion fractures. Our hypothesis was that such an approach would be a useful addition to the existing techniques. First, a 3D model of the acromion was printed, then an osteosynthesis plate was pre-bent to fit the exact shape and curve of the acromion. We tested this technique and present reports on five patients, three with os acromiales and two with acromial fractures. We followed these patients during their rehabilitation and evaluated them using the Constant–Murley and the Disabilities of the Arm, Shoulder and Hand scores.

**Results:**

In every case the fracture or non-union healed. If the surgery was performed before additional damage (such as an impingement syndrome) occurred, we saw that the patient’s pain completely disappeared. This new technique also has other advantages because the surgeon can prepare the entire operation in advance, which reduces the duration of surgery. Another advantage of using a 3D model is that it can also be used to inform the patient and the surgical team about the planned operation.

**Conclusion:**

This new technique using a preoperative patient-customized plate is a good alternative for use in open reduction and internal fixation, particularly if the patient has no other conditions.

## Background

An os acromiale results from non-union of the three ossification centres in the acromion. Under normal conditions, the pre-acromion, the meso-acromion and the meta-acromion should fuse by union of these centres. This normally occurs in adolescents between 15 and 18 years old (Abboud et al., 2006; Atoun et al., 2012; Barbier et al., 2013; Boehm et al., 2003; Frizziero et al., 2012; Harris et al., 2011; Ortiguera and Buss, 2002; Pagnani et al., 2006; Peckett et al., 2004; Sahajpal et al., 2007; Spiegl et al., 2015; Yammine, 2014). There are two competing hypotheses about the aetiology of non-fusion: some researchers believe that it is a consequence of a genetic defect, while others believe that it results from mechanical stress during the development of the acromion.

Os acromiale is uncommon. Several studies have investigated its prevalence, reporting varying frequencies, although most describe a frequency of 1%–15% (Ortiguera and Buss, 2002; Spiegl et al., 2015; Yammine, 2014) or 1%–30% (Harris et al., 2011; Sahajpal et al., 2007). Of these cases, 33%–62% show bilateral involvement (Harris et al., 2011; Ortiguera and Buss, 2002; Sahajpal et al., 2007; Yammine, 2014). Higher rates of os acromiale have been noted in Africans and men (Ortiguera and Buss, 2002; Sahajpal et al., 2007).

The method for treatment of a symptomatic os acromiale depends on its type, the severity of the pain and the preference of the surgeon, and there is no gold standard. If an operation is necessary, there are several options. The most frequently used procedures are open or arthroscopic excision of smaller os acromiales, arthroscopic decompression and open reduction and internal fixation (ORIF) of larger os acromiales.

The first approach is the open or arthroscopic excision of the unstable fragment. This operation has the highest success rate if it is performed on a pre-acromion. However, excision of a larger fragment will cause instability and often persistent postoperative deltoid dysfunction (Atoun et al., 2012; Boehm et al., 2003; Ortiguera and Buss, 2002; Pagnani et al., 2006; Peckett et al., 2004; Sahajpal et al., 2007; Spiegl et al., 2015).

The next option is an arthroscopic sub-acromial decompression. With this technique, the surgeon removes some bone from the anterior site of the acromion to create space for the underlying supraspinatus. This is a commonly used technique to treat impingement syndromes if the os acromiale is stable (Harris et al., 2011). However, this procedure has one major disadvantage: it is a salvage operation that does not treat the underlying cause of the condition, so relapses are very common (Ortiguera and Buss, 2002; Sahajpal et al., 2007).

The last option is an ORIF. The purpose of this technique is to set the fracture, reduce the inferior slope of the os acromiale, compress it and fix it. There are several possible techniques for ORIF. The first technique is fixing the os acromiale using two K-wires drilled parallel from anterior to posterior through the defect (Spiegl et al., 2015). A figure-of-eight tension band can be applied to strengthen the fixation. This approach does not provide the most solid fixation and there is a risk of hardware irritation and migration (Barbier et al., 2013; Harris et al., 2011). The second common technique is the use of cannulated screws drilled over two small guide wires (Spiegl et al., 2015), using either the classic short treated spongiosa screws or headless compression screws such as the Acutrak® (Acumed LLC, Hillsboro, Oregon 97,124, USA). The screws provide a more rigid fixation than the K-wires, but there is often hardware irritation at the insertion point of the deltoid muscle on the acromion, prompting hardware removal at a later stage (Harris et al., 2011). Another disadvantage is that these screws are sometimes too big to fit in a very thin acromion (see case 3 below). Both techniques can also be combined with cranial cerclage for better stability (Peckett et al., 2004).

Fracture of the acromion has effects similar to those of os acromiale. Acromion fractures of the scapula are rare: just 1% of all fractures are scapular fractures and of these only 8%–10% involve the acromion (Anavian et al., 2009; Tucek, 2017). These fractures usually occur as sequelae of high-energy injuries concomitantly with fractures of the ipsilateral glenoid, neck and body of the scapula (Almzayyen et al., 2013). Classification of these fractures using current systems is very difficult. Indications for operative management include symptomatic non-union, displaced fractures or acromion fractures associated with other lesions of the superior shoulder suspensory complex (Hillewaere and Dierickx, 2014).

We have developed an alternative technique based on using a pre-bent osteosynthesis plate originally designed for the distal clavicle to achieve the correct fixation and reduction of the acromion. We tested this technique on cadaver specimens and concluded that it provides a stronger repair than cannulated screws and cerclage (Loomans et al., 2017). Several manufacturers market pre-contoured superior locked plates for distal complex clavicular fractures (Andersen et al., 2011). The plate we used, from DePuy Synthes (4528 Zuchwil, Switzerland), is well-documented to be a reliable fixation device for complex distal clavicular fractures with or without extra fixation to the coracoid process (Almzayyen et al., 2013; Vaishya et al., 2017). In our method, the osteosynthesis plate is pre-bent using a three-dimensional (3D) model of the patient’s acromion as a template.

We present five retrospective case reports, including three patients with os acromiale and two with acromial fracture.

## Methods

### Preoperative: 3D printing of the acromion model

**Fig. 1:**
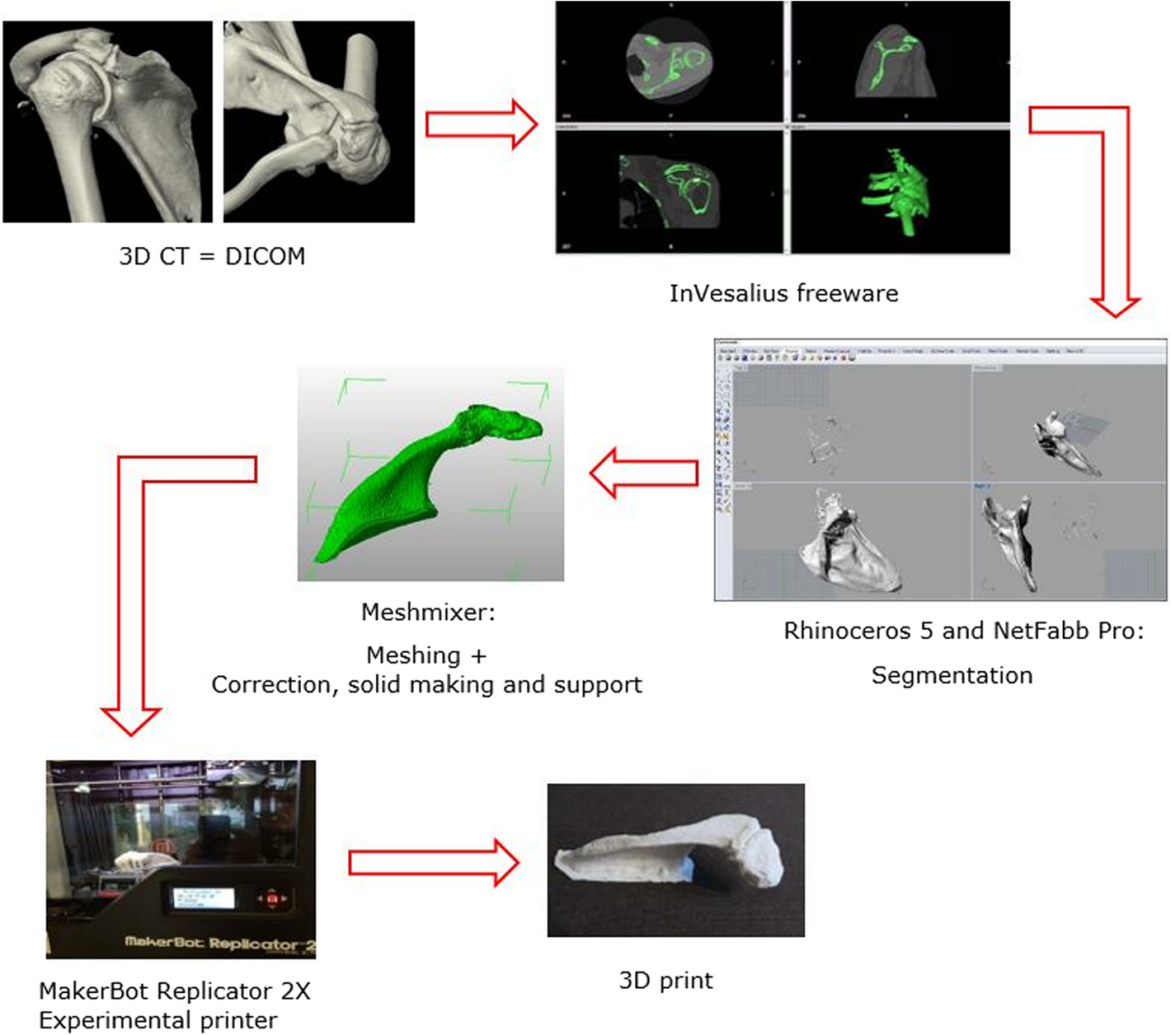
Process of 3D printing. A 3D CT scan (DICOM file) was converted into a file the printer could recognise using InVesalius freeware. Rhinoceros 5 and Netfabb Pro were used to segment the desired area of interest, after which Meshmixer was used to extract a surface from this area (meshing) and for further processing of the image (correction, solid assembly and support). A 3D print was made using the MakerBot Replicator 2X Experimental printer

There is a high interindividual variability in the shape of the acromion (El-Din and Ali, 2015). Therefore, there are no specific plates available for the acromion and we had to adapt an osteosynthesis plate designed for the distal clavicle. To do this, a 3D model of the patient’s acromion was necessary. Several steps were necessary to obtain a 3D print (Fig. 1).

The first step was to obtain a 3D model of the patient’s acromion by taking a 3D computed tomography (CT) scan of the shoulder using a 320-slice CT scanner (Aquilion One; Toshiba). The protocol used in our patients is standard in our institute (Table 1).

**Table 1:** Protocol for 3D–printing

Position	Patient in supine position
	Shoulder in the middle
	Humerus in exo-rotation
Field of view	From the acromion to the tip of the scapula
Resolution	Axial: ST 0.5/GAP
	0.25 bone kernel
	Cor/Sag 2/2 bone recons

*ST* slice thickness, *Cor* coronal, *Sag* sagittal

The scan results were delivered in a data imaging and communications in medicine (DICOM) format. This is a two-dimensional image and must be converted from a DICOM file to a file that the printer can recognise, usually a standard tessellation language (STL) file, using InVesalius freeware (Marro et al., 2016). We used Rhinoceros 5 and Netfabb Pro for the segmentation of the desired area of interest and to provide identification annotations. When the segmentation was finished, we used Meshmixer to extract a surface from the isolated area and to correct for artefacts in the image. This process is called meshing. It was very important to keep comparing the original scan and the newly made 3D image to ensure that all proportions were respected. Meshmixer was also used for the further processing of the image. This involved assembly of the solid and inserting supports for the prints. The supports were necessary to prevent collapse during printing according to the fused deposition modelling (FDM) printing system.

The printer used in this study was the MakerBot Replicator 2X Experimental printer. This is an FDM printer and uses acrylonitrile butadiene styrene (ABS) or polylactic acid (PLA), both filaments of thermoplastic polymer. Once the model was printed, post-processing was needed to remove the supports.

### Preoperative: Pre-bending of the distal clavicle plate

**Fig. 2:**
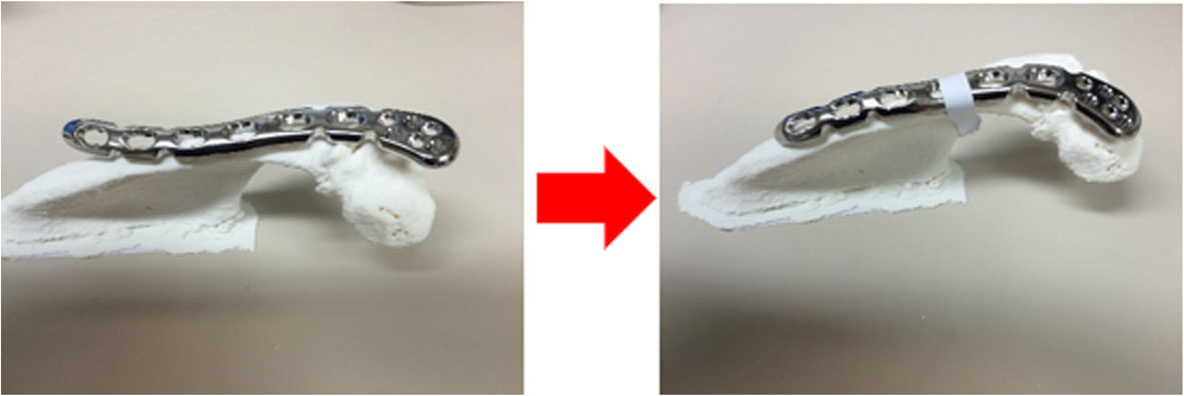
Bending the osteosynthesis plate based on the 3D print. A correction was made to lift the anterior acromion and to prevent further impingement on the rotator cuff

For these operations, we made off-label use of small (three or four holes) 3.5-mm locking compression plates (LCP), distal clavicle reconstruction plates manufactured by DePuy Synthes. These plates incorporate Combi holes: one side of the Combi hole is threaded and can be used for fixation using locking screws, which provides angular stability. The other side of the hole is the dynamic compression unit, which is unthreaded and was used for dynamic compression of the non-union using cortical screws. To bend these plates, we used standard plate benders provided by DePuy Synthes to adjust all types of 3.5-mm LCP distal clavicle reconstruction plates (Synthes, 2009). In adjusting the plate, we considered the correction needed to lift the anterior acromion to prevent further down-sloping of the acromion and subsequent impingement on the rotator cuff (Fig. 2).

### Operation technique

This technique can be used both for treatment of an os acromiale and for stabilisation of a fracture of the acromion.

**Fig. 3:**
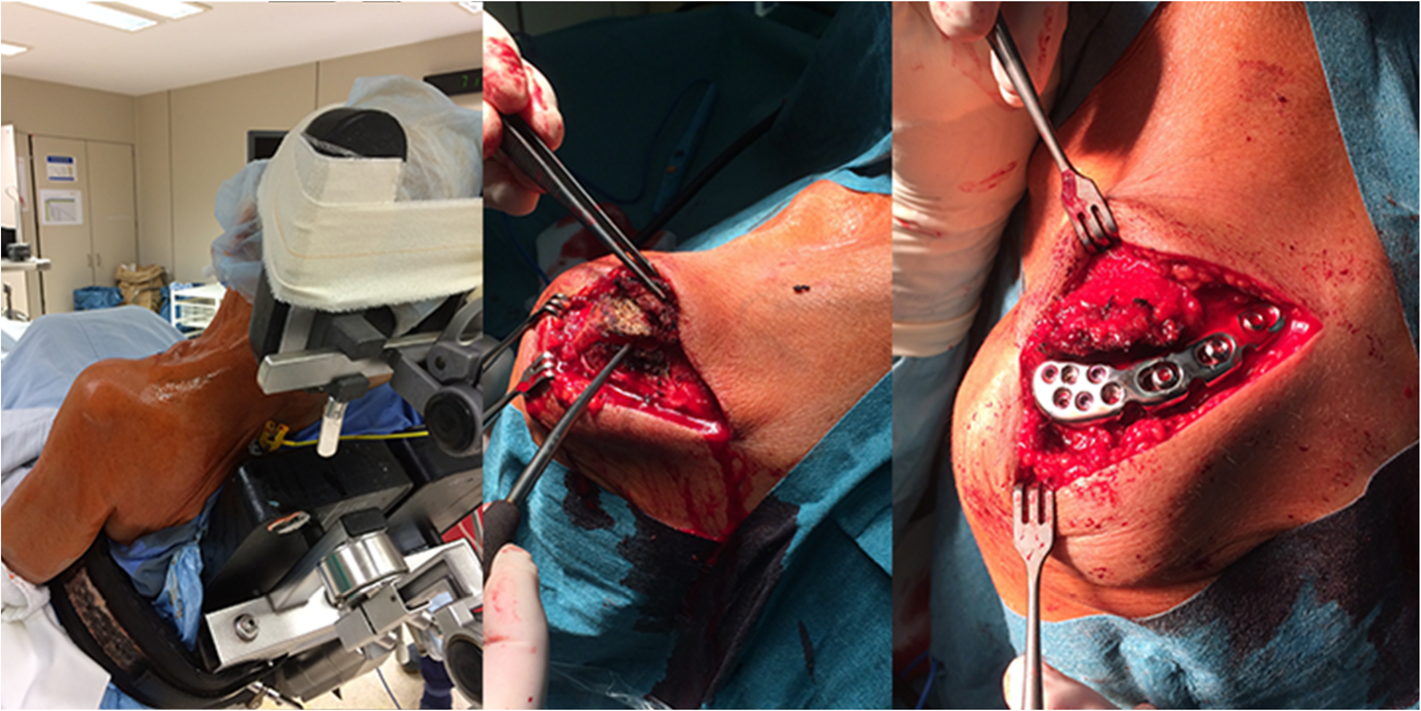
Course of the surgery. The patient was placed in the beach-chair position without a tourniquet (left). A longitudinal incision was made along the scapular spine to visualise the entire os acromiale or acromial fracture (middle). The osteosynthesis plate was fixed anteriorly with angle-stable screws and posteriorly with angle-stable screws and at least one cortical compression screw to compress the fracture (right)

The patient was placed in the beach-chair position without a tourniquet. A longitudinal incision was made along the scapular spine to make the entire acromiale visible. The pre-bent osteosynthesis plate was put in place to check that the pre-bending of the plate was adequate. In cases of pseudarthrosis or os acromiale, the plate was then removed so an osteotomy of 1 mm could be performed on each side of the fracture. This step was necessary for proper bone healing, but could be omitted if the fracture had been treated without previous pseudarthrosis. The plate was subsequently replaced and first fixed anteriorly with six 2.7-mm angle-stable screws with a length 1 mm less than the measured depth of the acromion. In this way, perforation of the lower sub-acromial surface could be prevented. Posteriorly, beyond the fracture line, we used a minimum of one cortical compression screw to compress the fracture. Angle-stable 3.5-mm screws were used for further fixation (Fig. 3).

If an osteotomy was performed, the remnants of the bone were pulverized and used as a bone graft. Otherwise, the bone graft was harvested from the iliac crest. Once the bone graft was applied, the wound was closed. The position of the plate and screws was verified by radiography.

To evaluate this technique, the five patients (Table 2) who were treated were evaluated using the Constant–Murley and the Disabilities of the Arm, Shoulder and Hand (DASH) scores.

**Table 2:** General information about the patients

Initials	General info	FU	Injury	First/second operation	Illustration
PW	• Male • 48 y	13 M	Bilateral os acromiale, only the left side was symptomatic	First operation	
RF	• Male • 42 y	6 M	Os acromiale on the left side, with signs of impingement	Third operation	
TR	• Female • 67 y	6 M	Os acromiale and treated PASTA type tear of RC, on the left side	Second operation	
VL	• Male • 28 y	5 M	Fracture of the acromion and coracoid process on the right side	First operation	
VR	• Male • 60 y	/	Fracture of the acromion, ribs and coracoid process on the right side	First operation	

*FU* follow-up (from operation until last postop), *y* years old, *M* months, *RC* rotator cuff

The Constant–Murley score is a system that uses scores to evaluate pain, daily activities, strength and range of motion of the shoulder using tests and a questionnaire that result in a score on a scale of 0–100. Scores of 0–55, 56–70, 71–85 and 86–100 points indicate “poor”, “moderate”, “good” and “excellent” outcomes, respectively (Constant and Murley, 1987).

DASH is a scoring system that consists of two components: the disability component and an optional module concerning high-performance sport/music and work. The optional module detects difficulties that are present in some work environments or among professional athletes/performing artists, but that are undetected in daily activities. All activities are scored 1 to 5, with 1 indicating “no problem” and 5 indicating “impossible”. The eventual DASH score is calculated using the formula $\left[\frac{\left( sum\  of\ n\  responses\right)}{n}-1\right]\ x\ 25$, which results in a score of 0–100. The lower the score, the better the outcome (Institute for Work & Health, 2006).

## Discussion of the five cases

### Case 1

Patient PW was a 48-year-old male triathlete who had bilateral os acromiale, but only the left side was locally symptomatic. The acromion was sensitive on palpitation and after swimming. All impingement signs were negative and the ultrasound and magnetic resonance imaging (MRI) showed marked oedema at the os acromiale but did not reveal any rotator cuff tendinopathy. The patient was first treated with local conservative therapy. This included physical therapy with ice and an infiltration of the os acromial joint with Kenacort and Xylocaine 2%. This gave immediate pain relief for a few hours. Conservative treatment was unsuccessful, meaning that an operation was necessary. In this case, nine screws (2.7- and 3.5-mm) were used. Six anterior angle-stable screws were necessary. Posteriorly two cortical screws (one of which was used for compression) and two angle-stable locking screws were used. Because of the congenital or primary pseudarthrosis, an osteotomy of 1 mm was performed on each side of the osseous non-union including the cranial osteophytes. These bone remnants were pulverized and replaced between or lateral to the plate and the osteotomy site. The control radiography showed correct positioning.

### Case 2

Patient RF was a 42-year-old man who had a left side os acromiale with an impingement syndrome. He was first treated by a colleague orthopaedic surgeon, who performed three surgeries. The first was an arthroscopic limited decompression, followed by fixation with K-wires and tension band wiring. Because of an infection and perforation of the osteosynthesis materials, they were removed and replaced by two Acutrak screws. However, a monitoring CT showed loosening of these screws. The patient continued to suffer from a loss of strength and pain at night. As an alternative approach, an osteosynthesis using a distal clavicle osteosynthesis plate was performed. Fixation was achieved using cortical and angle-stable locking screws (4 × 10 mm and 2 × 12 mm). One compression screw was used. A bone graft was harvested from the left iliac crest and applied at the osteotomy site.

### Case 3

Patient TR was a 67-year-old woman who had a left-side os acromiale together with a rotator cuff tear, a type of partial articular supraspinatus tendon avulsion and a biceps hourglass impingement. The first operation (2009) was an arthroscopic decompression, biceps tenotomy and rotator cuff repair. Because of recurrent pain with documented impingement syndrome, an osteosynthesis with an osteosynthesis plate was performed. An osteotomy of 1 mm was performed either side of the non-union. Fixation was achieved using cortical and angle-stable screws (14 or 16 mm) and one compression screw was used. Her acromion was so small that the usual procedure of inserting 3.5-mm hollow screws was not possible. The bone remnants were pulverized and used as a bone graft.

### Case 4

Patient VL was a 28-year-old homeless male. He was admitted to the emergency room after a fall while cycling. He complained of pain in his right shoulder and an impaired range of motion. An X-ray showed a fracture of the acromion and the coracoid process with a slight dislocation of the fragments. This fracture was classified as an S2c fracture according to the lateral scapula suspension system (LSSS) introduced by Lambert et al. (2013). These fractures together with concomitant ligamentous lesions of the acromioclavicular (AC) ligament caused instability of his shoulder, which made an operation necessary.

During surgery, compression of the fracture was achieved using one cortical and two angle-stable screws. Anterior fixation was achieved using six angle-stable screws (10 or 12 mm). The coracoid fracture was treated percutaneously with a hollow screw as described by Hillewaere and Dierickx (2014), with the exception that in our case radioscopic rather than arthroscopic monitoring of the reduction was used. After surgery, the patient was advised to stop smoking to avoid impairing the healing process.

### Case 5

Patient VR was a 60-year-old homeless male. He experienced a frontal collision on his motorcycle under the influence of alcohol. He had a history of alcohol, nicotine and heroin abuse. He complained about pain in his right shoulder. A CT showed fractures of the acromion, the coracoid process with an intra-articular glenoid component, the scapula tip and three rib fractures. These fractures could be classified according to the LSSS as S1b and S2c fractures, without including the concomitant scapular tip fracture and three rib fractures (Lambert et al., 2013). This scapular fracture was similar to that of patient VL, and there was also a rupture of the AC ligaments, which caused instability.

This fracture was set using an osteosynthesis plate and six anterior 12-mm angle-stable locking screws and three posterior compression screws. The AC ligaments were repaired and the coracoid fracture was treated the same way as that of patient VL. A monitoring X-ray showed good positioning of the osteosynthesis plate and screws.

## Results

The postoperative radiographs and CTs showed a complete fusion of the fracture/os acromiale in all five cases. Below, each patient is discussed in detail (Table 3).

**Table 3:** Evaluation of the patients

	Preoperative imaging	Postoperative imaging	Constant-Murley (CM) and DASH (D) score
PW M 48 y			CM: 85/100 ➔ “Good” D: 13.33/100
RF M 42 y			CM: 55/100 ➔ “Poor” D: 52.5/100
TR F 67 y			CM: 36/100 ➔ “Poor” D: 76.39/100
VL M 28 y			CM: 90/100 ➔ “Excellent” D: 6.82/100
VR M 60 y			CM: / D: / ➔ Homeless: lost for follow-up

*M* male, *F* female, *y* years old, *DASH* Disabilities of the Arm, Shoulder and Hand

### Case 1

During his first postoperative evaluation 4 months after surgery, patient PW showed full passive mobility and had no pain from the plate or wounds. However, he complained of pain during active elevation in endo-rotation. Radiography showed clearly that two anterior screws were too prominent. The patient was first treated with a test infiltration, but the pain returned after 3 h. The screws were removed and replaced by two smaller screws (12 mm) during a second operation performed as day surgery.

After 13 months, the patient had a Constant–Murley score of 85/100, which was classified as good to excellent, and a DASH score of 13.33/100. He also completed the two optional modules of the DASH questionnaire, one concerning hobbies and one concerning work. The score for the first module was 68.75/100 and for the second module 18.75/100. He swims, runs and rides his bicycle.

### Case 2

The rehabilitation went very well for patient RF and he had good passive mobility and wound healing. Radiography showed a good position of the plate and some incorporation of the bone graft. At 2 and a half months after surgery, his anterior deltoid function remained impaired and impingement tests were still positive. Mobility had stabilised at 60° exo-rotation and 100° abduction. At 4 months after surgery, the signs of impingement persisted. An infiltration of corticosteroids was given, after which the pain disappeared and abduction normalised. At the 6-month follow-up he had a Constant–Murley score of 55/100, classified as poor. The low score was mainly because of his constant pain and limited daily activities, although his mobility was very good. He had a DASH score of 52.5/100. The reason for this rather poor score was the same as for the Constant–Murley score, i.e., the remaining supraspinatus tendinopathy. A revision arthroscopic decompression/bursectomy with shaving of the bursal side of the supraspinatus tendon and removal of one slightly perforating screw was performed.

### Case 3

Patient TR regained good passive mobility and radiography showed correct positioning of the osteosynthesis plate without loosening. However, the patient still experienced pain from the persisting supraspinatus tendinopathy 4 months postoperatively. Six months after the operation, her Constant-Murley score was 36/100, which is rated as poor. This low score was because of severe pain and limited daily activities. Active mobility was moderate. She had a DASH score of 76.39/100, which confirmed the conclusions from the Constant–Murley score.

### Case 4

Patient VL had a smooth rehabilitation. Physiotherapy was started twice a week after 1 month. Five months after the operation, he had a Constant–Murley score of 90/100 and a DASH score of 6.82/100. These scores are similar to those that can be expected in perfectly healthy people.

### Case 5

Patient VR was lost to follow up because he remained homeless, but was reported by his general practitioner to be doing well. We will not discuss him any further because we cannot compare him with the others.

## Discussion

The aim of this study was to assess whether this new technique using a patient-customized plate is an acceptable alternative to the standard of care for os acromiale and acromial fractures. Our study showed that this technique gives good results when used to treat acromial fractures. The results for treatment of os acromiale were more variable. However, it must be noted that all os acromiales and acromial fractures healed completely. Therefore, we believe that this technique is a valid alternative to existing techniques for treating os acromiale or acromial fracture.

Other surgical techniques sometimes do not provide proper healing of the acromion; e.g., when the acromion is so thin that insertion of a hollow screw is impossible. In this situation, an osteosynthesis plate can be used instead. As with every ORIF approach, the technique of using a patient-customized plate may have the disadvantage of causing hardware irritation (Harris et al., 2011). Another limitation is its higher cost (Martelli et al., 2016). We compared the costs of our technique and of an ORIF using cannulated screws and tension band. The total costs of the DePuy Synthes materials necessary for the hollow-screw fixation were approximately 540 euros, while those for our technique were approximately 900 euros. Our technique is obviously more expensive even when the costs for the digital preparation and in-house 3D printing were not considered. In Belgium, however, most of these costs for implants are refunded; thus, the extra cost of the LCP plate and screws to the patients was only 105.10 euros. Further investigations are necessary to obtain an accurate estimate of all costs and not just those of the materials.

In this case series, we used an FDM printer that had some advantages: the material used can be easily changed and it is low cost compared with other printers, making it affordable for an individual surgeon or hospital. However, its 3D printing is a slow process and the layer thickness is at least 0.15 mm (AlAli et al., 2015). A disadvantage is that the model obtained cannot be sterilised; therefore, it cannot be used in the operating theatre. However, the FDM printer was adequate for our purposes because we did not need a high level of detail and the pre-bending of the osteosynthesis plate could be done in advance; therefore, only the plate needed to be sterilised.

The use of 3D printing can be seen as an additional step in the overall surgical protocol, but it provides several advantages. The first advantage relates to the preparation of the surgical approach: the 3D model shows the underlying lesions in a tactile and visual way, which provides a better understanding of complex fractures or deformities. The best options for potential difficulties of the surgical intervention can be considered even before the operation has started. This results in a more complete preparation for the surgical team and a less invasive intervention for the patient (Bosc et al., 2017; Zheng et al., 2016; Martelli et al., 2016). In addition, fewer instruments are needed. The second advantage of 3D printing is that the surgeon can operate more efficiently and with more confidence because of their improved preparation. The surgeon will know more and need to measure less, which leads to a shorter duration of the intervention (Bosc et al., 2017; Martelli et al., 2016). The duration of anaesthesia will also be shorter, which is safer for the patient (Cronskär, 2014). The third advantage of the 3D models is better communication between surgeon, radiologist and nursing staff, resulting in a smoother procedure (Bosc et al., 2017; Thomas et al., 2016).

The 3D printing can be outsourced to specialised firms, but this may be very expensive. We suggest “in-house printing” in the clinic itself. This is much cheaper and faster and perhaps even more accurate. If the 3D scans are processed by an outside company, they are processed by engineers who do not have sufficient knowledge of the anatomy of the acromion to maintain the right proportions, meaning that time may be lost in cases of trauma. If the printing is done “in house”, the normal 6-week delivery time for a 3D model can be reduced to 48 h, which makes this technique applicable in trauma settings such as our patients (Bosc et al., 2017). However, because we selected only very rare and complex cases, our case number was limited, and further investigation in a multi-centre setting is recommended to test efficiency and safety.

## Conclusion

The non-union healed completely in all five of our cases; therefore, we can conclude that this technique using a patient-customized plate achieves the correct fixation and reduction of the acromion to allow complete repair of the lesion. Although we admit that some of our patients were still in pain, it is important to note that these patients have a long medical history. For example, they have had previous operations and have other related symptoms such as a rotator cuff tear. We conclude that if there is no condition present except a symptomatic os acromiale, this technique can provide appropriate treatment. This was confirmed by the results of the two fracture cases that were treated and by the results of one of the patients with os acromiale (PW).

## Abbreviations

2D: Two Dimensional
3D: Three Dimensional
ABS: Acrylonitrile Butadiene Styrene
AC: Acromioclavicular
CT: Computed Tomography
DASH: Disabilities of the Arm, Shoulder and Hand
DICOM: Data Imaging and COmmunications in Medicine
FDM: Fused Deposition Modelling
LCP: Locking Compression Plates
LSSS: Lateral Scapula Suspension System
MRI: Magnetic Resonance Imaging
ORIF: Open Reduction and Internal Fixation
PASTA: Partial Articular Supraspinatus Tendon Avulsion
PLA: Polylactic Acid
STL: Standard Tessellation Language
